# Supplementary material for: The association between community solidarity and adoption of public health preventive measures during the COVID-19 pandemic in a cross-sectional, multi-national sample
Source: PLoS One. 2025 Jun 24;20(6):e0324234. doi: 10.1371/journal.pone.0324234 (PMC12186886; doi:10.1371/journal.pone.0324234)
Supplement: S1 Tables — (DOCX) [file pone.0324234.s001.docx]

**Supporting Information 1**

**S1 Table 1: Estimated dates of widespread COVID-19 vaccine availability for countries with residents included in study sample**

| **Country** | **Estimated date that COVID-19 vaccine was available to all adults** |
| --- | --- |
| Australia | 19 August 2021 |
| Canada | 1 May 2021 |
| China | 1 July 2021 |
| Ecuador | 1 July 2021 |
| France | 31 May 2021 |
| Germany | 6 May 2021 |
| India | 1 May 2021 |
| Japan | 21 June 2021 |
| Malaysia | 1 May 2021 |
| Mexico | 1 June 2021 |
| New Zealand | 10 May 2021 |
| Philippines | 15 October 2021 |
| Russia | 17 February 2021 |
| South Korea | 17 June 2021 |
| Thailand | 6 June 2021 |
| United Kingdom | 17 June 2021 |
| United States of America | 18 April 2021 |

**S1 Table 2. Results of logistic regression predicting social distancing during COVID-19**

| Predictor | Odds Ratio (95% CI) |
| --- | --- |
| Reported positive feelings of solidarity | **2.05 (1.46, 2.89)** |
| Month of survey completion (ref = January 2022)  January 2021  February 2021  March 2021  April 2021  May 2021  June 2021  July 2021  August 2021  September 2021  October 2021  November 2021  December 2021 | 0.46 (0.10, 2.04)  0.47 (0.10, 2.34)  0.97 (0.19, 5.10)  0.29 (0.06, 1.42)  0.75 (0.13, 4.50)  1.05 (0.40, 2.77)  --  3.23 (0.53, 19.82)  0.85 (0.32, 2.25)  1.32 (0.67, 2.61)  1.08 (0.28, 4.22)  1.21 (0.13, 10.81) |
| Completed survey after COVID-19 vaccine was made available to all adults in respondent’s country of residence | 1.08 (0.28, 4.14) |
| Region of residence (ref = West)  East | 1.10 (0.59, 2.05) |
| Age category (ref = 18 to 29)  30 to 39  40 to 49  50 and over | 1.24 (0.72, 2.12)  **2.95 (1.18, 7.38)**  **2.64 (1.04, 6.70)** |
| Sex (ref = Male)  Female | **1.53 (1.10, 2.15)** |
| Education (ref = More than 12 years)  Less than 10 years  10-12 years | 0.70 (0.30, 1.64)  **0.60 (0.38, 0.96)** |
| Household composition (ref = Living alone)  Living with family  Living with roommates  Other | **1.73 (1.03, 2.92)**  1.52 (0.78, 2.98)  4.65 (0.55, 39.34) |
| Employment (ref = Full-time employed)  Part-time employed  Self-employed  Retired  Student  Not employed but not student  Caregiver or other | 0.76 (0.34, 1.67)  0.41 (0.17, 1.02)  0.38 (0.08, 1.71)  1.00 (0.64, 1.58)  0.41 (0.16, 1.07)  0.47 (0.16, 1.38) |
| Financial situation in past 6 months (ref = Decreased)  Stayed the same  Increased  Don’t know | 1.21 (0.80, 1.83)  0.80 (0.49, 1.31)  1.49 (0.71, 3.14) |
| Receiving welfare at the time of survey completion | 0.82 (0.54, 1.22) |
| Urban-rural status (ref = Rural area)  Rural-urban fringe  Urban area | 0.76 (0.41, 1.43)  1.05 (0.69, 1.58) |
| Reported ever having a vaccine against influenza | 1.20 (0.84, 1.70) |

*Note.* N=1346 participants who had complete data on outcome and all predictors; **bold font** indicates a statistically significant odds ratio.

**S1 Table 3: Results of logistic regression predicting staying home from work/school during COVID-19**

| Predictor | Odds Ratio (95% CI) |
| --- | --- |
| Reported positive feelings of solidarity | 1.30 (0.97, 1.74) |
| Month of survey completion (ref = January 2022)  January 2021  February 2021  March 2021  April 2021  May 2021  June 2021  July 2021  August 2021  September 2021  October 2021  November 2021  December 2021 | **0.10 (0.03, 0.28)**  **0.18 (0.05, 0.57)**  0.45 (0.15, 1.37)  **0.11 (0.03, 0.36)**  **0.22 (0.06, 0.79)**  0.72 (0.33, 1.58)  0.79 (0.12, 5.34)  1.30 (0.40, 4.22)  **0.30 (0.14, 0.64)**  1.27 (0.75, 2.16)  1.11 (0.37, 3.34)  -- |
| Completed survey after COVID-19 vaccine was made available to all adults in respondent’s country of residence | 1.58 (0.63, 3.95) |
| Region of residence (ref = West)  East | 0.71 (0.42, 1.20) |
| Age category (ref = 18 to 29)  30 to 39  40 to 49  50 and over | 1.08 (0.69, 1.70)  0.98 (0.55, 1.72)  1.25 (0.67, 2.31) |
| Sex (ref = Male)  Female | 0.92 (0.68, 1.24) |
| Education (ref = More than 10 years)  Less than 10 years  10-12 years | 0.70 (0.34, 1.42)  1.21 (0.78, 1.88) |
| Household composition (ref = Living alone)  Living with family  Living with roommates  Other | 1.57 (0.96, 2.58)  0.96 (0.49, 1.88)  0.57 (0.11, 2.93) |
| Employment (ref = Full-time employed)  Part-time employed  Self-employed  Retired  Student  Not employed but not student  Caregiver or other | 1.24 (0.65, 2.37)  1.74 (0.76, 4.00)  **4.98 (1.18, 20.95)**  **2.77 (1.83, 4.20)**  **3.79 (1.42, 10.12)**  1.09 (0.43, 2.73) |
| Financial situation in past 6 months (ref = Decreased)  Stayed the same  Increased  Don’t know | 0.77 (0.54, 1.11)  0.67 (0.43, 1.07)  0.63 (0.33, 1.21) |
| Receiving welfare at the time of survey completion | **0.68 (0.48, 0.97)** |
| Urban-rural status (ref = Rural area)  Rural-urban fringe  Urban area | 0.79 (0.46, 1.36)  0.77 (0.54, 1.10) |
| Reported ever having a vaccine against influenza | 1.13 (0.83, 1.53) |

*Note.* N=1346 participants who had complete data on outcome and all predictors; **bold font** indicates a statistically significant odds ratio.

**S1 Table 4: Results of logistic regression predicting skipping an event one wanted to attend during COVID-19**

| Predictor | Odds Ratio (95% CI) |
| --- | --- |
| Reported positive feelings of solidarity | **1.40 (1.08, 1.80)** |
| Month of survey completion (ref = January 2022)  January 2021  February 2021  March 2021  April 2021  May 2021  June 2021  July 2021  August 2021  September 2021  October 2021  November 2021  December 2021 | **0.36 (0.13, 0.96)**  0.53 (0.17, 1.62)  0.36 (0.12, 1.07) 0.34 (0.11, 1.04)  0.87 (0.25, 3.02)  0.81 (0.39, 1.69)  0.85 (0.13, 5.59)  2.12 (0.69, 6.53)  **0.35 (0.17, 0.70)**  1.03 (0.63, 1.70)  0.45 (0.18, 1.12)  0.70 (0.16, 3.19) |
| Completed survey after COVID-19 vaccine was made available to all adults in respondent’s country of residence | 1.56 (0.65, 3.75) |
| Region of residence (ref = West)  East | 1.03 (0.66, 1.63) |
| Age category (ref = 18 to 29)  30 to 39  40 to 49  50 and over | **1.74 (1.15, 2.62)**  **1.99 (1.16, 3.44)**  **2.18 (1.21, 3.95)** |
| Sex (ref = Male)  Female | 1.19 (0.92, 1.54) |
| Education (ref = More than 10 years)  Less than 10 years  10-12 years | 0.64 (0.33, 1.22)  0.91 (0.63, 1.30) |
| Household composition (ref = Living alone)  Living with family  Living with roommates  Other | **1.66 (1.07, 2.56)**  1.34 (0.76, 2.36)  1.14 (0.31, 4.24) |
| Employment (ref = Full-time employed)  Part-time employed  Self-employed  Retired  Student  Not employed but not student  Caregiver or other | 1.07 (0.57, 1.99)  0.59 (0.28, 1.25)  0.63 (0.19, 2.12)  1.03 (0.72, 1.46)  0.66 (0.29, 1.52)  0.85 (0.35, 2.05) |
| Financial situation in past 6 months (ref = Decreased)  Stayed the same  Increased  Don’t know | 1.01 (0.74, 1.37)  0.83 (0.56, 1.23)  0.93 (0.56, 1.57) |
| Receiving welfare at the time of survey completion | 1.01 (0.74, 1.38) |
| Urban-rural status (ref = Rural area)  Rural-urban fringe  Urban area | 0.85 (0.52, 1.39)  0.74 (0.55, 1.01) |
| Reported ever having a vaccine against influenza | **1.44 (1.11, 1.87)** |

*Note.* N=1346 participants who had complete data on outcome and all predictors; **bold font** indicates a statistically significant odds ratio.

**S1 Table 5: Results of logistic regression predicting masking in public during COVID-19**

| Predictor | Odds Ratio (95% CI) |
| --- | --- |
| Reported positive feelings of solidarity | **3.31 (1.94, 5.66)** |
| Month of survey completion (ref = January 2022)  January 2021  February 2021  March 2021  April 2021  May 2021  June 2021  July 2021  August 2021  September 2021  October 2021  November 2021  December 2021 | 1.16 (0.16, 8.52)  2.39 (0.21, 27.67)  --  0.47 (0.06, 4.03)  1.04 (0.10, 11.09)  1.07 (0.30, 3.79)  --  1.09 (0.17, 7.00)  1.91 (0.50, 7.21)  1.82 (0.72, 4.64)  2.13 (0.24, 18.91)  -- |
| Completed survey after COVID-19 vaccine was made available to all adults in respondent’s country of residence | 0.92 (0.16, 5.24) |
| Region of residence (ref = West)  East | 2.74 (1.12, 6.70) |
| Age category (ref = 18 to 29)  30 to 39  40 to 49  50 and over | 2.34 (0.88, 6.16)  2.72 (0.74, 10.02)  1.43 (0.41, 5.01) |
| Sex (ref = Male)  Female | **2.46 (1.50, 4.02)** |
| Education (ref = Less than 10 years)  10-12 years  More than 12 years | 0.43 (0.16, 1.14)  0.83 (0.44, 1.58) |
| Household composition (ref = Living alone)  Living with family  Living with roommates  Other | 1.39 (0.58, 3.32)  0.83 (0.28, 2.43)  1.49 (0.15, 15.12) |
| Employment (ref = Full-time employed)  Part-time employed  Self-employed  Retired  Student  Not employed but not student  Caregiver or other | 0.64 (0.22, 1.82)  0.37 (0.12, 1.15)  **--**  0.86 (0.42, 1.76)  0.30 (0.09, 1.04)  0.72 (0.14, 3.70) |
| Financial situation in past 6 months (ref = Decreased)  Stayed the same  Increased  Don’t know | 0.55 (0.29, 1.07)  **0.45 (0.20, 0.99)**  0.44 (0.17, 1.16) |
| Receiving welfare at the time of survey completion | 0.87 (0.47, 1.62) |
| Urban-rural status (ref = Rural area)  Rural-urban fringe  Urban area | 1.50 (0.59, 3.80)  1.50 (0.83, 2.69) |
| Reported ever having a vaccine against influenza | **2.16 (1.24, 3.76)** |

*Note.* N=1346 participants who had complete data on outcome and all predictors; **bold font** indicates a statistically significant odds ratio.

**S1Table 6: Results of logistic regression predicting willingness to get the COVID-19 vaccine**

| Predictor | Odds Ratio (95% CI) |
| --- | --- |
| Reported positive feelings of solidarity | 1.38 (0.99, 1.89) |
| Month of survey completion (ref = January 2022)  January 2021  February 2021  March 2021  April 2021  May 2021  June 2021  July 2021  August 2021  September 2021  October 2021  November 2021  December 2021 | **0.07 (0.02, 0.28)**  **0.09 (0.02, 0.39)**  **0.03 (0.01, 0.12)**  **0.11 (0.02, 0.47)**  **0.10 (0.02, 0.51)**  **0.06 (0.02, 0.16)**  0.15 (0.01, 1.84)  0.42 (0.08, 2.17)  0.68 (0.19, 2.43)  0.57 (0.29, 1.10)  1.41 (0.30, 6.75)  0.78 (0.09, 7.01) |
| Completed survey after COVID-19 vaccine was made available to all adults in respondent’s country of residence | **1.26 (1.08, 1.45)** |
| Region of residence (ref = West)  East | **0.32 (0.15, 0.66)** |
| Age category (ref = 18 to 29)  30 to 39  40 to 49  50 and over | 1.31 (0.79, 2.19)  1.43 (0.70, 2.94)  1.51 (0.68, 3.32) |
| Sex (ref = Male)  Female | 1.02 (0.74, 1.40) |
| Education (ref = More than 10 years)  Less than 10 years  10-12 years | 1.06 (0.49, 2.30)  1.04 (0.67, 1.60) |
| Household composition (ref = Living alone)  Living with family  Living with roommates  Other | 0.90 (0.53, 1.53)  0.78 (0.41, 1.52)  0.27 (0.07, 1.03) |
| Employment (ref = Full-time employed)  Part-time employed  Self-employed  Retired  Student  Not employed but not student  Caregiver or other | 0.84 (0.41, 1.74)  1.30 (0.45, 3.75)  0.44 (0.12, 1.72)  1.30 (0.85, 1.97)  0.43 (0.17, 1.13)  1.62 (0.44, 5.94) |
| Financial situation in past 6 months (ref = Decreased)  Stayed the same  Increased  Don’t know | 0.99 (0.68, 1.44)  1.19 (0.74, 1.92)  1.26 (0.65, 2.43) |
| Receiving welfare at the time of survey completion | **0.61 (0.42, 0.86)** |
| Urban-rural status (ref = Rural area)  Rural-urban fringe  Urban area | 1.38 (0.73, 2.60)  1.33 (0.91, 1.93) |
| Reported ever having a vaccine against influenza | **1.86 (1.34, 2.57)** |

*Note.* N=1346 participants who had complete data on outcome and all predictors; **bold font** indicates a statistically significant odds ratio.
